# Spectral EMG Changes in Cervical Dystonia Patients and the Influence of Botulinum Toxin Treatment

**DOI:** 10.3390/toxins9090256

**Published:** 2017-08-23

**Authors:** S. W. R. Nijmeijer, E. de Bruijn, R. Verhagen, P. A. Forbes, D. J. Kamphuis, R. Happee, M. A. J. Tijssen, J. H. T. M. Koelman

**Affiliations:** 1 Department of Neurology and Clinical Neurophysiology, Academic Medical Center, 1105 AZ Amsterdam, The Netherlands; s.w.nijmeijer@amc.uva.nl (S.W.R.N.); r.verhagen@amc.uva.nl (R.V.); 2 BioMechanical Engineering, Delft University of Technology, 2628 CD Delft, The Netherlands; edodebruijn@gmail.com (E.d.B.); p.a.forbes@tudelft.nl (P.A.F.); r.happee@tudelft.nl (R.H.); 3 Department of Neurology, Reinier de Graaf Groep, 2625 AD Delft, The Netherlands; Daan.Kamphuis@rdgg.nl; 4 Department of Neurology AB 51, University of Groningen, 9713 GZ Groningen, The Netherlands; m.a.j.de.koning-tijssen@umcg.nl

**Keywords:** cervical dystonia, botulinum toxin, EMG, autospectral analysis, muscle selection

## Abstract

Botulinum toxin (BoNT) injections in the dystonic muscles is the preferred treatment for Cervical Dystonia (CD), but the proper identification of the dystonic muscles remains a challenge. Previous studies showed decreased 8–14 Hz autospectral power in the electromyography (EMG) of splenius muscles in CD patients. Cumulative distribution functions (CDF’s) of dystonic muscles showed increased CDF_10_ values, representing increased autospectral powers between 3 and 10 Hz, relative to power between 3 and 32 Hz. In this study, we evaluated both methods and investigated the effects of botulinum toxin. Intramuscular EMG recordings were obtained from the splenius, semispinalis, and sternocleidomastoid muscles during standardized isometric tasks in 4 BoNT-naïve CD patients, 12 BoNT-treated patients, and 8 healthy controls. BoNT-treated patients were measured 4–7 weeks after their last BoNT injections and again after 11–15 weeks. We found significantly decreased 8–14 Hz autospectral power in splenius muscles, but not in the semispinalis and sternocleidomastoid muscles of CD patients when compared to healthy controls. CDF_10_ analysis was superior in demonstrating subtle autospectral changes, and showed increased CDF_10_ values in all studied muscles of CD patients. These results did not change significantly after BoNT injections. Further studies are needed to investigate the origin of these autospectral changes in dystonia patients, and to assess their potential in muscle selection for BoNT treatment.

## 1. Introduction

Dystonia is a syndrome characterized by sustained or intermittent involuntary muscle contractions, leading to debilitating abnormal postures and twisting movements [1]. In cervical dystonia (CD), the most common form of primary dystonia, the neck muscles are primarily involved [2]. The first line treatment for CD is botulinum toxin (BoNT) injection in the dystonic muscles [3]. For an optimal treatment effect, proper selection of the dystonic muscles is essential [4,5]. Unfortunately, no specific tools are available to reliably identify dystonic muscles, and discriminate them from healthy compensating muscles [6]. In a previous study we obtained intramuscular electromyography (EMG) recordings to investigate the autospectra of the EMG envelope and intermuscular coherences as possible discriminative tools to identify dystonic muscles [7]. The most discriminating finding was an 8–14 Hz autospectral peak that was measured in the splenius capitis (SPL) muscles of almost all healthy controls during isometric contraction, but that was absent in the majority of the dystonic SPL muscles in patients. In a subsequent study we developed an isometric setup so that EMG recordings could be obtained during standardized, reproducible isometric tasks [8]. In analyzing the collected data we developed a new analysis method, capable of demonstrating more subtle autospectral changes in dystonic muscles. Cumulative distribution functions (CDF) provides a measure for the relative distribution of autospectral power. In this study, CDF was used to demonstrate an increase in power between 3 and 10 Hz (CDF_10_ value) relative to the power between 10 and 30 Hz in dystonia patients [9]. Higher CDF_10_ values were found on a group level in the SPL, the sternocleidomastoid (SCM), and the semispinalis capitis (SESP) muscles of CD patients when compared to equivalent muscles in healthy controls. This represents a power shift towards the lower frequency band (3–10 Hz) in these muscles. This pilot study, however, had several important limitations that we aim to address in the current study. An important limitation was that surface EMG electrodes were used, and electric crosstalk between the anatomically closely related neck muscles might have impaired the accurate identification of individual dystonic muscles. Another important limitation in all of the above-described studies was that all patients had already been treated with BoNT injections. BoNT injections cause muscle atrophy, decrease the EMG amplitude [10], influence muscle fiber velocity [11], and might reduce the median frequency of EMG autospectra [12]. All patients were measured at least 3 months after their previous BoNT treatment, but BoNT might still have had some effect after that period [12]. The precise effects of BoNT on the above-described autospectral changes are unknown, and therefore the influence of previous BoNT treatments on these results cannot be excluded. In the current study, we obtained intramuscular EMG recordings during standardized isometric tasks. Autospectra and CDF’s were evaluated in 4 BoNT-naïve patients, 12 BoNT-treated patients, and 8 healthy controls. All BoNT-treated patients were measured twice. The first measurement was 4–7 weeks after their last BoNT injections, and the second measurement was after 11–15 weeks, when most clinical BoNT effects are expected to have worn off [13]. The first goal was to investigate 8–14 Hz autospectral peaks and CDF_10_ values in our improved setup in CD patients and controls. The second goal was to investigate the possible confounding effect of BoNT on these results.

## 2. Results

### 2.1. Study Population

Twelve BoNT-treated patients (5 males and 7 females) aged between 46 and 74 years (mean age ± SD, 63 ± 8.9 years), and 4 BoNT-naïve patients (1 male and 3 females) aged between 44 and 63 years (mean age ± SD, 55 ± 8.0 years) participated in this study. In addition, 8 healthy controls aged between 41 and 65 years (mean age ± SD, 51.5 ± 9.9 years) were recruited. For patients, the duration of their symptoms ranged from 3 to more than 40 years in the BoNT-treated patients (median; IQR, 8.5 years; 4.0–20.0 years), and from 2 to 20 years in BoNT-naïve patients (median; IQR, 10.5 years; 2.75–19.0 years). In the BoNT-treated patients, the mean TWSTR score was 29.6 (SD: 11.24). In BoNT-naïve patients the mean TWSTR score was 31.25 (SD: 13.25). For the BoNT-treated patients, the first measurement ranged from 29 to 45 days (median; IQR, 32 days; 30–37.5 days) after the previous BoNT treatment, and the second measurement ranged from 80 to 105 days (median; IQR, 96 days; 91–99 days) after the previous BoNT treatment. Clinical characteristics of the individual patients are presented in Table 1. All BoNT-treated patients received BoNT injections according to their usual treatment scheme tailored by their treating neurologist before participating in this study. Patient 07 received onabotulinumtoxinA (Botox^®^) injections, and all other BoNT-treated patients were treated with abobotulinumtoxinA (Dysport^®^) injections.

### 2.2. Maximal Force and Torque during Maximal Voluntary Muscle Contractions (MVC) Tasks

The means of the maximal horizontal rearward force, and maximal left and right rotational torque produced by patients and controls during the MVC tasks are shown in Table 2. The maximal delivered forces and torques were on average higher in healthy controls when compared to patients, although these differences were not statistically significant. 

### 2.3. Task Performance

In healthy controls, on average 4.9 of the 6 trials were completed successfully. In the BoNT-treated patients, on average 2.9 of the 6 trials were completed successfully during the first measurement, and 3.4 of the 6 trials were completed successfully during the second measurement. The BoNT-naïve patients completed 3.3 of the 6 trials successfully. 

### 2.4. Raw EMG Inspection

In most CD patients, the amplitude of the EMG signals was generally lower when compared to healthy controls, possibly reflecting a lower level of muscle contraction (see discussion). Nine EMG recordings from individual muscles during one of the measurements were excluded because of artefacts or needle displacements. In two patients (numbers 7 and 10), large artefacts were observed in the EMG of multiple muscles during several trials. As a result, the complete first measurement of patient number 10 had to be excluded from further analysis. For patient number 7, the second repetition of each trial from the first measurement had to be excluded. 

### 2.5. EMG Spectral Analysis: Pooled Autospectra

**Figure 1:**
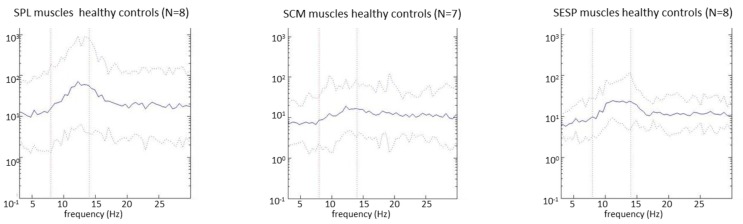

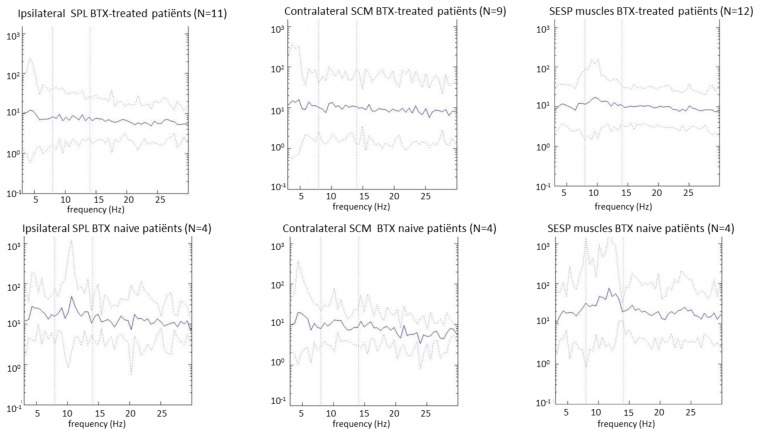
Pooled autospectra of the rectified electromyography (EMG) between 3 and 30 Hz. Solid line: mean of the log-transformed autospectra. Dotted line: 95% confidence intervals. In BoNT-treated patients the autospectra from the second measurements are shown, when most BoNT effects are expected to have worn off. Similar autospectra were seen during the first measurement.

In Figure 1, the pooled autospectra of the rectified EMG are displayed per group by the average of the log-transformed autospectra, including the 95% confidence intervals. In healthy controls, a clear 8–14 Hz peak is seen in the SPL muscles, and a more subtle 8–14 Hz peak is seen in the SESP muscles. This peak was absent in the SCM muscles. In the BoNT-treated muscles, no 8–14 Hz peaks were seen in any of the muscles. In the BoNT-naïve patients, a subtle 8–14 Hz peak was seen only in the SESP muscles. 

### 2.6. EMG Spectral Analysis: 8–14 Hz Area Under the Curve (AUC’s) in SPL Muscles

The 8–14 Hz AUC’s are shown in Table 3. The 8–14 Hz AUC was significantly lower in the ipsilateral (presumed dystonic) SPL muscles in BoNT-treated patients when compared to the SPL muscles of healthy controls, both during the first and during the second measurement. In the contralateral (non-dystonic) SPL muscles, the AUC was also lower when compared to the healthy controls, but the difference was only statistically significant during the first measurement (4–7 weeks after BoNT treatment). For the four BoNT-naïve patients, the 8–14 Hz AUC was lower only in the ipsilateral SPL muscles, but this difference was not statistically significant.

### 2.7. EMG Spectral Analysis: 8–14 Hz AUC’s in SCM and SESP Muscles

A trend was observed towards lower 8–14 Hz AUC’s in the contralateral (presumed dystonic) SCM muscles of BoNT treated patients when compared to healthy controls. This difference was only statistically significant during the first measurement. There were no significant differences in the 8–14 Hz AUC’s in the SESP muscles between patients and controls. 

### 2.8. Effect of BoNT Treatment on the 8–14 Hz AUC’s

**Figure 2:**
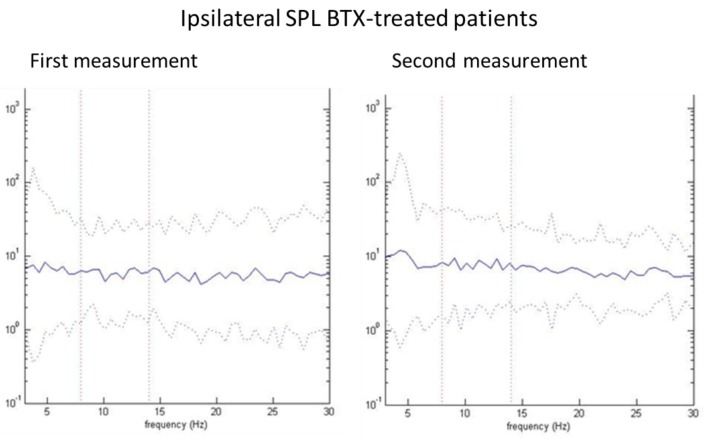
Pooled autospectra of the ipsilateral splenius capitis (SPL) in BoNT-treated patients during the first and second measurement. Solid line: mean of log-transformed rectified autospectra. Dotted line: 95% confidence interval.

Only in the contralateral (non-dystonic) SPL muscles, a statistically significant difference was observed between the first and the second measurement: the 8–14 Hz AUC was lower during the first measurement (*p* < 0.05). In the other muscles, no statistically significant differences were observed between the AUC’s from the first and second measurement. Figure 2 shows an example of the pooled autospectra of the ipsilateral SPL muscle in BoNT-treated patients during the first and during the second measurement.

### 2.9. Effect of Age, Gender and Tremor

There was no statistically significant correlation between age and the mean 8–14 Hz AUC’s. Furthermore, there was no significant difference in 8–14 Hz AUC’s between men or women. To evaluate the effect of tremor on the observed 8–14 Hz autospectral changes in the ipsilateral SPL muscles, we compared the 8–14 Hz AUC’s of the ipsilateral SPL muscles in patients with (N = 9), and without tremor (N = 3). The median 8–14 Hz AUC’s were comparable in patients with tremor (first measurement: 4.25; second measurement: 5.09) and patients without tremor (first measurement: 4.84; second measurement: 3.75). These differences were not statically significant.

### 2.10. CDF_10_ Analysis 

**Figure 3:**
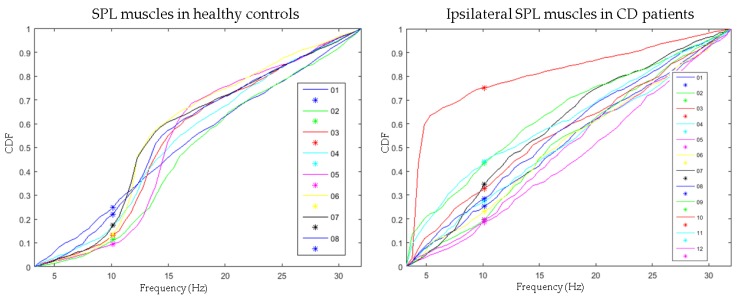
Plots of the CDF’s of the ipsilateral SPL muscles in Cervical Dystonia (CD) patients and the CDF’s of the SPL muscles in healthy controls during the second measurement. * CDF_10_ values. Individual lines: CDF plots of individual subjects. Note the deflected CDF curve of patient number 10. The corresponding autospectrum shows a high peak around 4–5 Hz responsible for this deflection and the raw EMG shows tremulous 4–5 Hz activity (data not shown). Excluding patient number 10 did not change the level of statistical significance.

The CDF_10_’s are shown in Table 4. The median CDF_10_ was higher in all of the muscles in patients when compared to the corresponding muscles in healthy controls. In the SPL muscles, the differences were statistically significant in both the ipsilateral and the contralateral SPL muscles in the BoNT-treated and the BoNT naïve patients. In the SCM muscles, the differences were statistically significant in the contralateral (dystonic) SCM muscles of the BoNT-treated patients and BoNT-naïve patients and in the ipsilateral (non-dystonic) SCM muscles only during the second measurement in the BTX-treated patients. In the SESP muscles, the differences were statistically significant in the BoNT-treated patients, but not in the BoNT-naïve patients. Plots of the CDF’s of the ipsilateral SPL muscles in patients, and the SPL muscles in healthy controls are shown in Figure 3. 

### 2.11. Effect of BoNT

There was no statistically significant difference in CDF_10_ values between the first and the second measurement in any of the muscles.

### 2.12. Effect of Age, Gender and Tremor

There was no significant correlation between age and the mean CDF_10_ values. Furthermore, there was no significant difference in CDF_10_ values between men or women. CDF_10_ values were higher in most, but not all muscles in patients with tremor (N = 9) as compared to patients without tremor (N = 3). However, after excluding all patients with tremor, the CDF_10_ values were still significantly higher in the ipsilateral SPL (*p* < 0.05), and contralateral SCM (*p* < 0.05) muscles in the remaining patients (N = 3) compared to healthy controls during both measurements. 

### 2.13. Diagnostic Potential to Discriminate Dystonia Patients from Healthy Controls 

#### 2.13.1. 8–14 Hz AUC’s

A receiver operating characteristic (ROC) curve was made using the 8–14 Hz AUC’s of all individual muscles of patients and controls. No cut-off existed with an acceptable sensitivity and specificity to discriminate patients from healthy controls, the area under the curve was 0.353.

#### 2.13.2. CDF_10_’s

**Figure 4:**
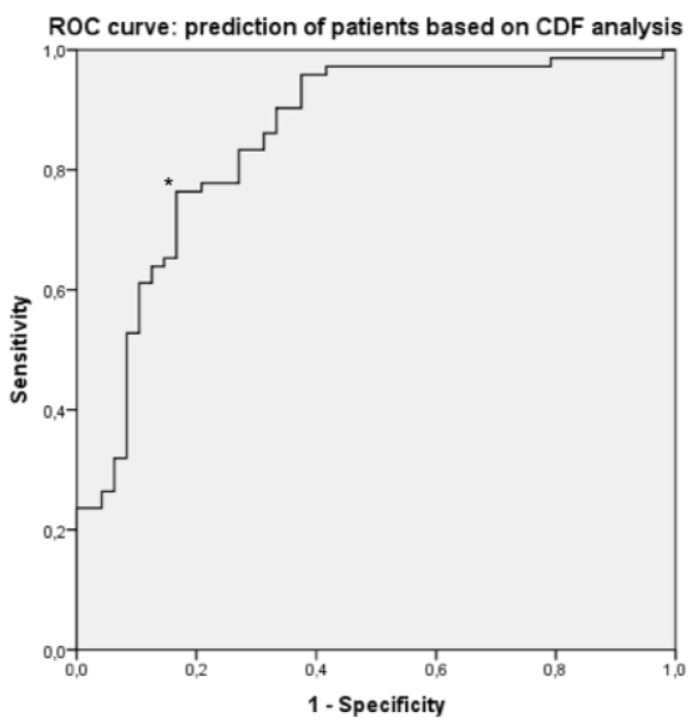
ROC (receiver operating characteristic) curve for identifying muscles that belong to CD patients based on different CDF_10_ values. (In a ROC curve the true positive rate (sensitivity) is plotted in function of the false positive rate (1-specificity) for different cut-off values (here, CDF_10_ values). * point on the ROC curve corresponding to a CDF_10_ value of 0.222. Test variable: CDF_10_ values. State variable: whether the muscles belong to a patient or a healthy control. The AUC of the ROC curve was 0.887.

**Figure 5:**
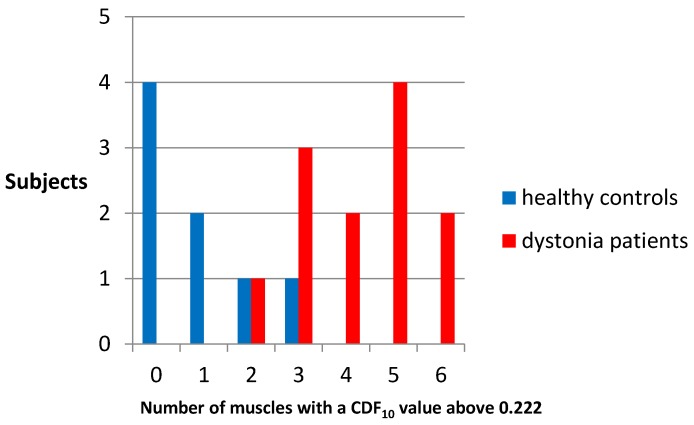
Number of muscles with a CDF_10_ above 0.222. Figure 5 illustrates the number of subjects (CD patients in red and healthy controls in blue) that have a certain number of muscles (specified on the *x*-axis) with CDF_10_ values above 0.222.

A ROC curve was made using the CDF_10_ values of all individual muscles of patients and controls. The area under the curve was 0.887. With a cut-off of 0.222, the sensitivity of CDF_10_ to identify patients was 0.773, and the specificity was 0.870 (Figure 4). In only one of the 8 healthy controls, and in 11 of the 12 BoNT-treated patients, at least half of the muscles had a CDF_10_ above 0.222 (Figure 5). 

## 3. Discussion

In this study, intramuscular EMG recordings were obtained during standardized isometric tasks to investigate 8–14 Hz autospectral changes, and CDF_10_ values in CD patients when compared to healthy controls. We found significant autospectral changes in CD patients that were more evident when CDF_10_ analysis was used. The autospectral changes could not be explained by the effect of BoNT injections. 

### 3.1. 8–14 Hz Autospectral Peaks in Healthy Controls

In line with previous studies [7,14], we found 8–14 Hz autospectral peaks in the SPL muscles of healthy controls, but not in CD patients. Several studies [7,14,15] have found evidence for synchronized activity at around 10–15 Hz in healthy neck muscles. These oscillations are hypothesized to facilitate common muscle synergies, and may originate from the reticular formation [15,16]. In limb muscles, oscillations are found in a higher (15–35 Hz) frequency band, and are linked to a corticospinal drive [17]. Blouin et al. therefore proposed that voluntary cortical activation of the neck motor neurons occurs through a relay in the reticular formation instead of a direct corticospinal projection [15]. In line with previous studies [7,14] we were unable to observe the same 8–14 Hz peaks in the autospectra of SCM muscles, and they were less evident in SESP muscles. It is possible that during our specific isometric experiments, the SPL muscles but not the SCM muscles were recruited into muscle synergies facilitated by 8–14 Hz oscillations. It could be speculated that the SCM and the SESP muscles would show similar 8–14 Hz autospectral peaks in slightly different postures, or when forces are produced in other directions. Another explanation might be that the absence of 8–14 Hz autospectral power in SCM muscles reflects differences in the innervation between cranial nerves (SCM) and spinal nerves (SPL and SESP).

### 3.2. Absence of 8–14 Hz Autospectral Peaks in Dystonia Patients

In dystonia patients, the 8–14 Hz autospectral peaks were absent in the SPL muscles during isometric tasks. If the hypothesis that these peaks reflect oscillations originating from the reticular formation is correct, their disappearance might point towards the involvement of the reticulospinal pathway in the pathophysiology of dystonia. Alternatively, in dystonia patients, there might be differences in muscle synergies resulting from longstanding changes in posture. Similarly, it is possible that slightly different posturing of the neck in patients during the experiments influenced the use of muscle synergies. In addition, suboptimal task performance in patients might have resulted in different patterns of muscle recruitment. It can, however, not be excluded that the observed autospectral differences are the result of other factors like muscle atrophy caused by multiple BoNT injections, or the generally lower degrees of muscle contraction in patients (see limitations). Finally, we have considered the explanation that normal 8–14 Hz autospectral peaks are masked or suppressed by a more prominent dystonic drive in the 4–7 Hz frequency band, as previously described in dystonia patients [7,14,18]. However, this seems unlikely as no clear 4–7 Hz peaks were seen in the pooled autospectra of patients.

### 3.3. CDF_10_ Analysis

CDF_10_ values were higher in all muscles in CD patients when compared to healthy controls, reflecting an increase in power between 3 and 10 Hz, relative to the power between 10 and 32 Hz. The higher CDF_10_ in patients could be explained by an increase in power of between 3 and 10 Hz, or by a decrease in power between 10 and 32 Hz. In this study we have found a decrease of 8–14 Hz autospectral power in patients when compared to the healthy controls. Because the 8–14 Hz autospectral peaks in the healthy controls were predominantly caused by power above 10 Hz (see Figure 1), the disappearance of these peaks in patients contributed to higher CDF_10_ values in patients. It is however unlikely that 8–14 Hz peaks are the only explanation for the higher CDF_10_ values because, unlike the higher CDF_10_ values, 8–14 Hz autospectral differences were not found in all muscles. An additional explanation might be increased activity in the 3–10 Hz band. EMG activity around 4–7 Hz has frequently been described in dystonia patients reflecting a dystonic drive, probably originating from the basal ganglia [7,14,19]. This drive is mostly seen in patients with rhythmic EMG activity, or even a visible dystonic tremor [7]. In this study, CDF_10_ values were indeed higher in most muscles in patients with tremor. However, it is unlikely that the 4–7 Hz dystonic drive, or a dystonic tremor are the only explanations for the higher CDF_10_ values as no clear 4–7 Hz peaks were visible in the pooled autospectra (Figure 1) that would explain the higher CDF_10_ values. Furthermore, significantly higher CDF_10_ values were also observed in patients without tremor. In conclusion, the higher CDF_10_ values in CD patients are probably the result of a combination of elevated power in the 4–7 Hz band, and reduced power in the 8–14 Hz band. Compared to autospectral analysis, CDF analysis is a more sensitive method to find subtle shifts in autospectral power in CD patients.

### 3.4. Effect of BoNT

To investigate the effect of BoNT on the 8–14 Hz autospectral changes and the higher CDF_10_ values, we measured the BoNT-treated patients twice. The first measurement was after 4–7 weeks, and the second measurement was 11–15 weeks after their previous BoNT treatment, when most BoNT effects are expected to have worn off [13]. Only in the contralateral (presumed non-dystonic) SPL muscles there was a statistically significant difference in 8–14 Hz autospectral power between the first and the second measurements. No statistically significant differences were observed in the other muscles, including the ipsilateral SPL muscles, where 8–14 HZ autospectral differences between patients and controls were the most evident. In the BoNT-naïve patients, no statistically significant differences in 8–14 Hz power were observed when compared to healthy controls. However, no definite conclusions can be drawn from this small group of BoNT-naïve patients (N = 4). For CD_F10_ values, no statistically significant differences were observed between the first and the second measurement. There was even a trend towards slightly higher CDF_10_ values during the second measurements. Furthermore, even in this small group of BoNT-naïve CD patients, significantly higher CDF_10_ values were observed when compared to healthy controls in the contralateral (presumed dystonic) SCM muscles and in the bilateral SPL muscles. Taken together: although we acknowledge that residual effects of BoNT can certainly not be ruled out, it seems unlikely that the observed low frequency autospectral differences are a pure BoNT effect. Therefore, it would be interesting to reproduce our findings in a larger group of BoNT naïve patients.

### 3.5. Task Performance

As could be expected, the number of successfully completed trials was higher in healthy controls. This confirms that dystonia patients have more difficulty in generating certain specific submaximal isometric forces. This could be the result of abnormal muscle activation patterns in dystonia patients during isometric tasks, as previously described [8]. Interestingly, in BoNT treated patients, the number of successfully completed trials was higher during the second measurement when effects of BoNT are expected to have worn off. This might be a training effect as patients were already familiar with the isometric tasks during the second measurement. 

### 3.6. Limitations

The most important limitation of this study was the small group of BoNT-naïve patients measured, as discussed above. An additional limitation might be the EMG artefacts that were observed in many subjects. These artefacts were caused by fluctuations in the currents exceeding the range of the EMG amplifier. Artefacts predominantly occurred when patients moved around between trials and were absent or limited in most of the relevant EMG segments. We excluded the trials with apparent artefacts in the relevant EMG segments. Finally, upon inspection of the raw EMG data, we noticed that the amplitude was generally higher in most muscles in the healthy controls. A higher amplitude is a measure of the magnitude of muscle force, suggesting a lower degree of muscle activation in patients [20]. Possibly, patients had more difficulty activating the correct muscles needed for specific tasks. Indeed, task performance was inferior in patients when compared to healthy controls. In addition, healthy controls might have had a higher overall degree of muscle contraction. This was reflected by higher produced maximal forces and torques by healthy controls, although these differences were not statistically significant. Another explanation for the lower EMG amplitude in patients might be the muscle atrophy caused by repeated BoNT treatment in patients. Finally, a lower amplitude in CD patients might have also have been caused by other factors, such as suboptimal needle placement, further away from the motor units [21]. The influence of these factors on the autospectral results is unclear, but to minimize the influence of differences in the autospectral power on our results, all autospectra were normalized before spectral analysis.

### 3.7. Future Perspectives and Potential Clinical Applications

CDF_10_ analysis is superior in demonstrating subtle low frequency autospectral changes in CD patients. Based on CDF_10_ values, we were able to discriminate patients from healthy controls with a sensitivity of 77%, and a specificity of 87%. Further studies are needed to investigate how adequate these tools are for the identification of dystonic muscles within patients. To determine the value of these autospectral changes for muscle selection before BoNT treatment, a large trial should be performed towards the effect of these methods on the outcome of BoNT treatment. Our results suggest that these autospectral changes do not change significantly after BoNT injections. If these methods can indeed also be applied in patients already treated with BoNT, it becomes more feasible to perform such a trial. Finally, frequency analysis of EMG recordings during different physiological and pathological conditions might provide more insight in the role of oscillatory drives towards neck muscles in healthy subjects and in dystonia.

## 4. Conclusions

In line with previous studies, we confirm the finding of 8–14 Hz autospectral peaks in the SPL muscles of healthy controls that are absent in dystonic SPL muscles in CD patients. Furthermore, we have shown that more subtle low frequency autospectral changes can be found in more muscles with CDF analysis, and that this method is superior in discriminating healthy controls from dystonia patients. Further studies are needed to investigate the origin of these autospectral changes, to determine how adequate these tools are for muscle selection for BoNT treatment, and to confirm that these methods can also be applied after patients have received BoNT.

## 5. Materials and Methods 

### 5.1. Study Population

Patients were recruited from a population of patients with CD, treated at the botulinum toxin outpatient clinic of the Academic Medical Center in Amsterdam (AMC). In addition, all neurologists connected to “DystonieNet” (a Dutch collaboration of neurologists specialized in dystonia, website: www.dystonienet.nl) were asked to inform eligible patients and ask them to participate in our study. Inclusion criteria were: (1) patients with idiopathic CD, and (2) a rotational component in the dystonic posturing. Exclusion criteria were: (1) fixed dystonia, or (2) secondary dystonia. Both BoNT-naïve patients, and those that were already receiving BoNT injections (BoNT-treated patients) were included. The BoNT-treated patients were measured twice: the first measurement was always between 4 and 7 weeks after the previous BoNT treatment, and the second measurement was always between 11 and 15 weeks after the previous treatment. Healthy controls were recruited using information posters in the AMC, and by asking the partners of patients. All subjects gave written, informed consent, and the study was approved by the medical ethics committee of the AMC (project code: 2013-134, date of approval: august 26^TH^, 2013). 

### 5.2. Clinical Examination

All subjects underwent a systematic neurological examination. For patients, dystonic symptoms were scored during their second visit using the Toronto Western Spasmodic Torticollis Rating Scale (TWSTRS, range: 0–87) [22].

### 5.3. Apparatus 

**Figure 6:**
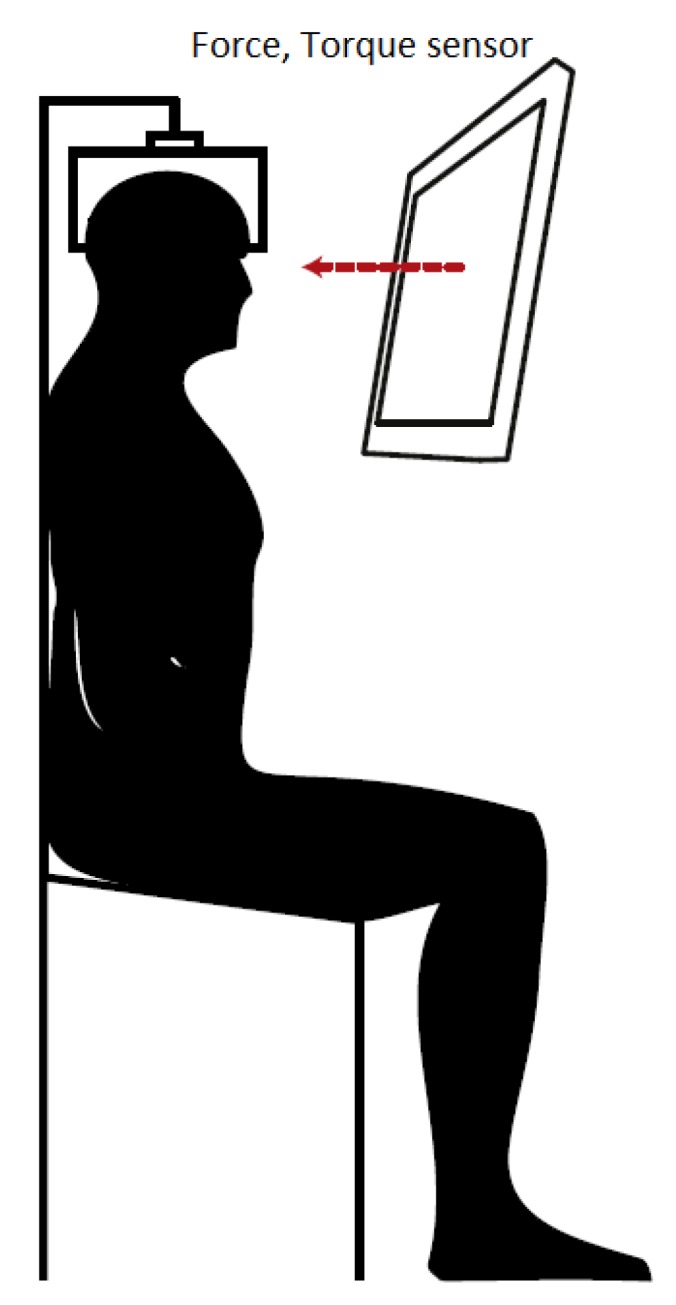
The isometric contraction device. The square represents a helmet on the subject’s head that is attached to the force/torque sensor.

Subjects were seated and fixed in an isometric device (Figure 6), with a tightly fitted cushioned helmet. They performed isometric neck muscle contraction tasks to generate horizontal force and rotational torque (spinal axis) that were measured by an overhead six axis load cell (MC3-6-500, AMTI Inc., Watertown, MA, USA). Force and torque signals were sampled at 2048 Hz. Subjects received visual feedback of the generated force and torque on a monitor in front of them. Visual feedback was presented through a custom made interface using Motek software (Motek Medical BV, Amsterdam, The Netherlands). After each task, a trigger pulse was sent to the EMG amplifier to mark the beginning and end of each trial in the EMG recordings. 

### 5.4. EMG Recordings

EMG was recorded and monitored using Micromed Systemplus Evolution (Mogliano Veneto, Treviso, Italy). Signals were amplified using a Micromed amplifier (SAM32RFO FC 32 channel headbox). Intramuscular EMG recordings were obtained bilaterally from the SCM, SPL, and SESP muscles using concentric needle electrodes. The needle electrodes were inserted by an experienced clinical neurophysiologist or by one of the investigators (S.N.) under supervision of an experienced clinical neurophysiologist. The wires of the electrodes were secured to the subject, using tape to prevent the needles from moving. Signals were sampled at 2048 Hz and monitored continuously during the experiment. If the signal was lost or the EMG activity was no longer visible during muscle contraction, the electrodes were checked and needles were re-inserted as necessary. 

### 5.5. Isometric Contraction Experiments

All tasks were explained to the subjects before the start of the experiment. Once secured in the isometric setup, subjects performed several practice runs to familiarize themselves with the device and the nature of the visual feedback. The experiment consisted of maximal and submaximal voluntary isometric contractions. First, subjects performed maximal voluntary muscle contractions (MVC) using neck muscles to generate head forces in one horizontal direction (rearward), and to generate left and right rotational torque. These MVC trials lasted five seconds in each direction, and were repeated twice. After the MVC trials, EMG needle electrodes were inserted bilaterally in the SPL, SCM, and SESP muscles for the subMVC trials. Patients now aimed to generate 20% of their MVC in the above mentioned directions. To reduce differences in task-performance between subjects, positive visual feedback was given when patients produced the target force/torque with a maximum error of 35%, and no concurrent force or torque greater than 35% of the target force/torque in any other direction. For example, if patients involuntarily produced a concurrent force greater than 35% of the target force in the horizontal plane during a rotation task, no positive feedback was given. After the target force/torque was reached, a timer started counting down from 8 s. In order to successfully complete the trial, subjects had to produce a stable force/torque, and remain within the prescribed limits for the full 8 s. All subjects were given several attempts of 30 s to successfully complete these trials. If the subjects were unable to complete a trial, they were instructed to maintain a steady force/torque as close as possible to the target force/torque for at least 8 s without making any further compensating movements. The sub-MVC trials were repeated at least twice in all directions. If no two successfully completed trials were available, the EMG recordings obtained during their two best attempts were used for further analysis.

### 5.6. Selection of Tasks and Muscles for Spectral Analysis

All muscles were analyzed during the task in which they were most likely to be active. The right SPL and left SCM were analyzed from the right rotational torque tasks and the left SPL, and the right SCM from the left rotational torque tasks. The SESP muscles were analyzed from the tasks in which subjects produced a horizontal rearward force. In patients, a distinction was made between the SCM and SPL muscles ipsilateral, and the SCM and SPL muscles contralateral to the direction of their dystonic posturing. The ipsilateral SPL and the contralateral SCM were most likely to be responsible for the rotational component of the dystonia (presumed dystonic muscles), and were therefore expected to show the most abnormalities specific for dystonia. 

### 5.7. EMG Visual Inspection

All raw EMG recordings were visually inspected and checked for reliable EMG activity and for unwanted artefacts. If no EMG activity was visible during muscle contraction, or if many artefacts were observed, the corresponding trials were excluded from further analysis. In most subjects, artefacts were observed in a limited number of EMG recordings. These were most likely caused by fluctuations in the EMG voltage exceeding the range of the EMG amplifier. These artefacts mainly occurred when subjects were moving in between tasks. If such artefacts were observed during the experiment, the corresponding trial was repeated. If significant artefacts were noticed after the experiment, the corresponding trial was excluded from further analysis. 

### 5.8. EMG Pre-Processing

EMG signals were processed offline using Vision Analyser 2.0 (Brain Products GmbH, Gilching, Germany). The raw bipolar EMG was notch filtered at 50 Hz, high pass filtered at 20 Hz (4th order zero-phase), low pass filtered at 750 Hz, and full wave rectified to obtain the EMG envelope. The last 0.5 s of each task were discarded because in many subjects the trigger pulse indicating the end of a trial created artefacts in the EMG data. Therefore, in each direction, two segments of 7.5 s were used for further analysis. To reduce the effects of variability in EMG amplitude between patients, each rectified EMG recording was normalized using the median of the amplitudes of the entire 7.5 s recording. The median was chosen here to minimize the influence of high amplitude discharges in the EMG. 

### 5.9. EMG Spectral Analysis

Low frequency characteristics of the EMG envelope were studied offline using Matlab (Matworks Inc., Natick, Massachusetts, USA) by calculating the autospectrum of the normalized rectified EMG recordings. For each 7.5 s trial, the autospectrum was calculated by averaging the discrete Fourier transformations of four non-overlapping 1.875 s segments, resulting in a frequency resolution of 0.5333 Hz. If two trials for one task were available, the autospectra were averaged over these trials. For group comparison, pooled autospectra were calculated using an average of the individual autospectra. Two measures were obtained from the individual autospectra using both of the absolute low-frequency powers and the CDF’s. Because the absolute low-frequency powers of the EMG envelope were skewed to the right, the autospectra were log-transformed. Subsequently, the power between 8 and 14 Hz was investigated by calculating the area under the curve (AUC) of the log-transformed autospectrum between 8 Hz and 14 Hz (more precisely 13.87 Hz because of frequency resolution restrictions). A second measure was obtained from the normalized CDF which was calculated to demonstrate the more subtle differences in the lower (3.2–32 Hz) frequency band [9]. The CDF was normalized to be zero at 3.2 Hz and one at 32 Hz, such that the CDF at 10 Hz (more precisely 10.13 Hz because of frequency resolution restrictions—from here on called CDF_10_) signified the power present in the lower (3.2–10 Hz) band as a ratio of the power over the full frequency band (3.2–32 Hz) band. 

### 5.10. Potential of 8–14 Hz AUC’s and CDF_10_ to Discriminate Dystonia Patients from Healthy Controls

To investigate the potential of 8–14 Hz AUC’s and CDF_10_ values to discriminate dystonia patients from healthy controls, ROC (receiver operating characteristic) curves were calculated using the ROC function in SPSS. The 8–14 Hz AUC’s and CDF_10_ values of all individual muscles were used as the test variable and the subject to which they belonged (patient or healthy control) was used as the state variable. The resulting ROC curves show the sensitivity and specificity for different CDF_10_ values, and 8–14 Hz AUC’s (represented by the different points on the curve) to discriminate CD patients from healthy controls. Based on this ROC curve, an optimal cut-off value was identified (value with the highest sensitivity and specificity) and subsequently the number of muscles above that cut-off value was compared between patients and controls.

### 5.11. Statistical Analysis

Clinical characteristics are presented using the mean and standard deviation (SD) for normally distributed data, and median and interquartile range (IQR) for non-normally distributed data. Due to the small numbers of patients included and presence of non-normally distributed variables, we used non-parametric statistics for the analysis of autospectral differences. For the SESP muscles in patients, and for all muscles in healthy controls, the results of the left and right muscles were first averaged. A Mann–Whitney U test was used to compare the 8–14 Hz AUC’s and the CDF_10_ values between the different muscles. To compare the AUC’s and CDF_10_ values between different measurements within subjects, the Wilcoxon signed rank test was used. Due to the exploratory nature of this study, we made no statistical corrections for multiple testing. To investigate the influence of age and gender, all subjects were grouped together, and the mean 8–14 Hz AUC’s and CDF_10_ values of all muscles were calculated for each subject. The influence of age was investigated using the Spearman rank correlation test, and the influence of gender was investigated using the Mann–Whitney U test. The independent samples t-test was used to compare the maximal produced force and torque during the MVC tasks between the subjects. All statistical analyses were performed using SPSS 22 (IBM, Armonk, New York, USA). 

## Figures and Tables

**Table 1:** Clinical characteristics. Pat. nr.: patient number. Patients 1–12 are the botulinum toxin (BoNT)-treated patients and patients 21–24 are the BoNT-naïve patients. ^1^ M: male F: female. ^2^ R: right; L: Left; ^3^ Numbers 1 to 3 indicate the degree of rotation, laterflexion, antecollis or retrocollis: 1: 0–15 degrees; 2: 15–30 degrees; 3: >30 degrees. ^4^ A: antecollis; R: retrocollis; NP: no antecollis or retrocollis present. ^5^ 0: no visible tremor 1: mild or intermittent tremor 2: pronounced tremor. ^6^ Total dosage of BoNT-injections during the previous treatment; MU: mouse units. N.A.: not applicable; B: onabotulinumtoxinA (Botox^®^) D: abobotulinumtoxinA (Dysport^®^).

Pat. nr.	Sex ^1^	Age	Dystonic Posturing			Tremor ^5^	TWSTR	Duration Symptoms (years)	BoNT Dosage (MU) and Type ^6^
			Rotation ^2,3^	Lateroflexion ^2,3^	Antecollis/Retrocollis ^3,4^				
01	F	51	L3	L2	NP	0	57.5	4	320 (D)
02	F	72	L1	R1	A1	1	36.75	36	190 (D)
03	M	67	L2	R1	NP	2	21	14	810 (D)
04	F	74	R2	R1	NP	1	14	4	240 (D)
05	F	65	R3	R1	NP	0	37.5	4	220 (D)
06	M	53	L2	R1	NP	1	20	20	410 (D)
07	F	61	R1	R1	NP	1	23.5	13	150 (B)
08	M	64	L2	L1	NP	0	26	>40	540 (D)
09	F	46	R1	R1	NP	1	35	4	240 (D)
10	F	66	L2	R1	A2	1	27	20	350 (D)
11	M	73	L2	R1	NP	1	29	3	640 (D)
12	M	64	R3	R1	A2	2	28	21	350 (D)
21	M	57	L1	L1	A1	1	33	2	N.A.
22	F	44	L1	R1	R1	1	16	20	N.A.
23	F	63	L3	R3	R3	2	48	16	N.A.
24	F	56	L1	L1	NP	1	28	5	N.A.

**Table 2:** Maximal force and torque. N: Newton; Nm: Newton Metre; SD: standard deviation; ns: not statistically significant compared to healthy controls.

Studied Subjects	Mean Maximal Horizontal (Rearward) Force (N)	Mean Maximal Left Rotational Torque (Nm)	Mean Maximal Right Rotational Torque (Nm)
**Healthy controls**	129.18 N (SD: 62.43)	6.25 Nm (SD: 3.40)	6.59 Nm (SD: 3.7)
**BoNT-treated patients** **First measurement**	93.36 N (SD: 36.24, ns)	4.04 Nm (SD: 2.40, ns)	4.66 Nm (SD: 2.49, ns)
**BoNT-treated patients** **Second measurement**	102.63 N (SD: 34.55, ns)	5.168 Nm (SD: 2.42, ns)	5.03 Nm (SD: 2.65, ns)
**BoNT-naïve patients**	88.10 N (SD: 28.52, ns)	4.92 Nn (SD: 1.77, ns)	4.86 Nm (SD: 2.34, ns)

**Table 3:** 8–14 Hz area under the curve (AUC’s) of the log-transformed autospectra. IQR: interquartile range. N = number of subjects used for analysis. * *p* values indicate the level of statistical significance of the difference in 8–14 Hz AUC’s compared to the corresponding muscles in healthy controls. Ns = not statistically significant. Results highlighted in bold represent statistically significant values. First measurement: 4–7 weeks after the previous BoNT treatment. Second measurement: 11–15 weeks after the previous BoNT treatment.

**8–14 Hz AUC’s**	**SPL Muscles in Healthy Controls**	**Ipsilateral (Presumed Dystonic) Splenius Capitis (SPL) Muscles**		
		**BoNT-Treated Patients, First Measurement**	**BoNT-Treated Patients, Second Measurement**	**BoNT-Naïve Patients**
Median 8–14 Hz AUC	8.151 (N = 8)	4.81 (N = 11, *p* < 0.001 *)	**5.02 (N = 12, *p* < 0.01)**	7.07 (N = 4, ns)
IQR	6.63–11.29	3.39–5.91	3.84–6.99	5.66–9.91
		**Contralateral (Presumed Non-Dystonic) SPL Muscle**		
		**BoNT-Treated Patients, First Measurement**	**BoNT-Treated Patients, Second Measurement**	**BoNT-Naïve Patients**
Median 8–14 Hz AUC		**5.19 (N = 11, *p* < 0.05)**	7.07(N = 11, ns)	9.19 (N = 4, ns)
IQR		4.70–7.06	6.29–9.06	7.61–12.54
	**SCM Muscles in Healthy Controls**	**Contralateral (Presumed Dystonic) SCM Muscles**		
		**BoNT-Treated Patients, First Measurement**	**BoNT-Treated Patients, Second Measurement**	**BoNT-Naïve Patients**
Median 8–14 Hz AUC	6.08 (N = 7)	**4.38 (N = 11, *p* < 0.05)**	4.58 (N = 9, ns)	5.79 (N = 4, ns)
IQR	4.80–7.26	3.76–5.84	4.18–8.37	5.31–6.87
		**Ipsilateral (Presumed Non-Dystonic) SCM Muscles**		
		**BoNT-Treated Patients, First Measurement**	**BoNT-Treated Patients, Second Measurement**	**BoNT-Naïve Patients**
Median 8–14 Hz AUC		6.65 (N = 10, ns)	6.84 (N = 12, ns)	5.32 (N = 4, ns)
IQR		5.97–7.90	4.92–7.98	4.40–8.18
	**SESP Muscles in All Subjects (Average between Left/Right Muscles)**			
	**Healthy Controls**	**BoNT-Treated Patients, First Measurement**	**BoNT-Treated Patients, Second Measurement**	**BoNT-Naïve Patients**
Median 8–14 Hz AUC	6.86 (N = 8)	5.97 (N = 11, ns)	6.13 (N = 12, ns)	8.97 (N = 4, ns)
IQR	6.50–8.31	4.89–7.45	5.11–7.34	6.37–10.82

**Table 4:** CDF_10_ values. CDF_10_: (Cumulative Distribution Function) at 10.13 Hz. IQR: interquartile range. N = number of subjects used for analysis. * *p* values indicating the level of statistical significance of the difference in CDF_10_ values compared to the corresponding muscles in healthy controls. Ns = not statistically significant. Results highlighted in bold represent statistically significant values. First measurement: 4–7 weeks after the previous BoNT treatment. Second measurement: 11–15 weeks after the previous treatment.

**CDF_10_**	**SPL Muscles in Healthy Controls**	**Ipsilateral (Presumed Dystonic) SPL Muscles**		
		**BoNT-Treated Patients, First Measurement**	**BoNT-Treated Patients, Second Measurement**	**BoNT-Naïve Patients**
Median CDF_10_	0.155 (N = 8)	0.278 (N = 11, *p* < 0.01 *)	**0.280 (N = 12, *p* < 0.001)**	**0.311 (N = 4 *p* < 0.01)**
IQR	0.118–0.209	0.256–0.354	0.207–0.413	0.253–0.480
		**Contralateral (Presumed Non-Dystonic) SPL Muscle**		
		**BoNT-Treated Patients, First Measurement**	**BoNT-Treated Patients, Second Measurement**	**BoNT-Naïve Patients**
Median CDF_10_		**0.227 (N = 11, *p* < 0.01)**	**0.319 (N = 11, *p* < 0.001)**	**0.285 (N = 4, *p* < 0.05)**
IQR		0.199–0.304	0.206–0.436	0.198–0.323
	**SCM Muscles in Healthy Controls**	**Contralateral (Presumed Dystonic) SCM Muscles**		
		**BoNT-Treated Patients, First Measurement**	**BoNT-Treated Patients, Second Measurement**	**BoNT-Naïve Patients**
Median CDF_10_	0.188 (N = 7)	**0.292 (N = 11, *p* < 0.001)**	**0.288 (N = 9, *p* < 0.01)**	**0.350 (N = 4, *p* < 0.01)**
IQR	0.129–0.194	0.241–0.339	0.240–0.417	0.266–0.492
		**Ipsilateral (Presumed Non-Dystonic) SCM Muscles**		
		**BoNT-Treated Patients, First Measurement**	**BoNT-Treated Patients, Second Measurement**	**BoNT-Naïve Patients**
Median CDF_10_		0.241 (N = 10, ns)	**0.296 (N = 12, *p* < 0.01)**	0.213 (N = 4, ns)
IQR		0.171–0.294	0.210–0.419	0.182–0.338
	**SESP Muscles in All Subjects**			
	**Healthy Controls**	**BoNT-Treated Patients, First Measurement**	**BoNT-Treated Patients, Second Measurement**	**BoNT-Naïve Patients**
Median CDF_10_	0.187 (N = 8)	**0.256 (N = 11, *p* < 0.05)**	**0.289 (N = 10, *p* < 0.01)**	0.264 (N = 4, ns)
IQR	0.142–0.215	0.202–0.273	0.226–0.341	0.146–0.359

## Supplementary Materials

The following are available online at http://www.mdpi.com/2072-6651/9/9/256/s1, Table S1: Muscles treated with BoNT.
