# Supplementary material for: Spectral EMG Changes in Cervical Dystonia Patients and the Influence of Botulinum Toxin Treatment
Source: Toxins (Basel). 2017 Aug 23;9(9):256. doi: 10.3390/toxins9090256 (PMC5618189; doi:10.3390/toxins9090256)
Supplement: Supplementary file 1 [file toxins-09-00256-s001.pdf]

Supplementary Materials: Spectral EMG Changes in Cervical Dystonia Patients and the Influence of Botulinum Toxin Treatment

S.W.R. Nijmeijer, E. de Bruijn, R. Verhagen, P.A. Forbes, D.J. Kamphuis, R. Happee, M.A.J. Tijssen and J.H.T.M. Koelman

Table S1. Muscles treated with BoNT.

Patient number	Muscles treated with BoNT	Dosage (MU) and type of BoNT
01	SPL (L), SCM (R), OBL (L)	320 (D)
02	SPL (B), SESP (B)	190 (D)
03	SPL (B), SCM (B), SESP (B), LSC (R), SCL (L), OBL (B)	810 (D)
04	SPL (B)	240 (D)
05	SPL (B), SCM(L)	220 (D)
06	SPL (L), SCM (R), LSC (L), SCL (L), OBL (L), TRP (L), PLT	410 (D)
07	SPL (R), SCM (l), LSC (B), OBL (R)	150 (B)
08	SPL (B), SCM (R), SESP (B), OBL (L), TRP (R)	540 (D)
09	SPL (B), SCM (B), TRP (R)	240 (D)
10	SPL (L), SCM (R), SESM (R), SCL (L), OBL (L)	350 (D)
11	SPL (L), SCM (R), SESP (B), LSC (R), OBL (R), TRP (R), PAR (R)	640 (D)
12	SPL (R), SCM (L), OBL (R)	350 (D)

(L): left, (R): right, (B): bilateral. MU: mouse units. OBL: obliquus capitis inferior, LSC: levator scapulae, SCL: Scalenus, TRP: trapezius, PLT: platysma, PAR: paraspinal muscles. B: onabotulinumtoxinA (Botox®) D: abobotulinumtoxinA (Dysport®)
